# N6-(2-Hydroxyethyl) Adenosine From *Cordyceps cicadae* Ameliorates Renal Interstitial Fibrosis and Prevents Inflammation via TGF-β1/Smad and NF-κB Signaling Pathway

**DOI:** 10.3389/fphys.2018.01229

**Published:** 2018-09-04

**Authors:** Rong Zheng, Rong Zhu, Xueling Li, Xiaoyun Li, Lianli Shen, Yi Chen, Yifei Zhong, Yueyi Deng

**Affiliations:** ^1^Department of Nephrology, Longhua Hospital, Shanghai University of Traditional Chinese Medicine, Shanghai, China; ^2^Chengjiaqiao Street Community Health Service Center, Shanghai, China

**Keywords:** N6-(2-hydroxyethyl) adenosine, *Cordyceps cicadae*, inflammation, renal interstitial fibrosis, unilateral ureteral obstruction

## Abstract

Renal interstitial fibrosis is characterized by inflammation and an excessive accumulation of extracellular matrix, which leads to end-stage renal failure. Our previous studies have shown that a natural product from *Cordyceps cicadae* can ameliorate chronic kidney diseases. N6-(2-Hydroxyethyl) adenosine (HEA), a physiologically active compound in *C. cicadae*, has been identified as a Ca^2+^ antagonist and an anti-inflammatory agent in pharmacological tests. However, its role in renal interstitial fibrosis and the underlying mechanism remains unclear. Here, unilateral ureteral obstruction (UUO) was used to induce renal interstitial fibrosis in male C57BL/6 mice. Different doses of HEA (2.5, 5, and 7.5 mg/kg) were given by intraperitoneal injection 24 h before UUO, and the treatment was continued for 14 days post-operatively. Histologic changes were examined by hematoxylin & eosin, Masson’s trichrome, and picrosirius red stain. Quantitative real-time PCR analysis, enzyme-linked immunosorbent assays, immunohistochemistry, and western blot analysis were used to evaluate proteins levels. And the results showed that HEA significantly decreased UUO-induced renal tubular injury and fibrosis. *In vivo*, HEA apparently decreased UUO-induced inflammation and renal fibroblast activation by suppression of the NF-κB and TGF-β1/Smad signaling pathway. *In vitro*, HEA also obviously decreased lipopolysaccharide-induced inflammatory cytokine level in RAW 264.7 cells and TGF-β1-induced fibroblast activation in NRK-49F cells by modulating NF-κB and TGF-β1/Smad signaling. In general, our findings indicate that HEA has a beneficial effect on UUO-induced tubulointerstitial fibrosis by suppression of inflammatory and renal fibroblast activation, which may be a potential therapy in chronic conditions such as renal interstitial fibrosis.

## Introduction

Chronic kidney disease occurs mainly as age-associated renal dysfunction, which affects approximately 35% of individuals over 75 years, and is accelerated by other chronic conditions such as diabetes mellitus (Jha et al., 2013; Hill et al., 2016). A consequence of chronic kidney disease is the transformation of activated interstitial fibroblasts into α-smooth muscle actin (α-SMA)-positive myofibroblasts (Neilson, 2006). The accumulation of these interstitial myofibroblasts and deposition of extracellular matrix (ECM) gives rise to renal interstitial fibrosis, a condition which contributes to renal functional loss (Liu, 2006; Farris and Colvin, 2012). The activation of interstitial fibroblasts in renal interstitial fibrosis is thought to be mediated by the gradual increase of growth factors and inflammatory cytokines, such as tumor necrosis factor-α (TNF-α), interleukin (IL)-1β, intercellular adhesion molecule-1 (ICAM-1), and monocyte chemotactic protein-1 (MCP-1) (Pang et al., 2010).

Transforming growth factor-β1 (TGF-β1) signaling is known to have a pivotal role in the activation of interstitial fibroblasts (Leask and Abraham, 2004; Lu et al., 2016; Higgins et al., 2018). The expression of TGF-β1 is increased in rats following unilateral ureteral obstruction (UUO) and is thought to be regulated by IL-10 (Meldrum et al., 2007; Sinuani et al., 2013). TGF-β stimulates the proliferation of fibroblasts and matrix accumulation through the binding of TGF-β type I and type II receptor complexes which in turn activates TβRI kinase, resulting in the phosphorylation and activation of Smad2 and Smad3 (Lan, 2011). Once activated, Smad2 and Smad3 translocate into the nucleus through forming oligomeric complexes with Smad4 and induce the expression of genes responsible for the production of the ECM (Meng et al., 2016). Smad7 negatively regulates Smad2 and Smad3, therefore, increased levels of Smad7 indicate a reduction in the production of ECM proteins.

Natural products are gaining interest as alternatives to conventional medicine for alleviating chronic conditions such as renal interstitial fibrosis (Wojcikowski et al., 2006). The fruiting bodies of *Cordyceps cicadae*, an entomopathogenic fungus, have been used as a natural medicine over the centuries to treat various conditions including childhood palpitations, epilepsy, and convulsions (Lu et al., 2015). The main active constituents of *C. cicadae* are adenosine, 3′-deoxyadenosine (cordycepin), and N6-(2-hydroxyethyl)-adenosine (HEA). The medicinal properties of adenosine and cordycepin include involvement in anti-inflammation, anticonvulsion, neuroprotection, antioxidation, antiangiogenesis, hypolipidemia, and immunoregulation (Latini and Pedata, 2001; Nakav et al., 2008; Zhou et al., 2008; Guo et al., 2010). HEA is a Ca^2+^ antagonist and anti-inflammatory agent that is associated with the control of cerebral and coronary circulation and is thought to possess sedative activity (Wang et al., 2013).

In a previous study, we found that extracts of *C. cicadae* can ameliorate chronic kidney disease simulated by a subtotal nephrectomy model in Sprague Dawley rats (Zhu et al., 2011). The depositions of type IV collagen and fibronectin were decreased and the expression of TGF-β1 and connective tissue growth factor were reduced following treatment with the extracts. In subsequent research, we investigated the influence of ergosterol peroxide, the major sterol produced by *C. cicadae*, in a fibroblast cell line (NRK-49F) stimulated by TGF-β1 (Zhu et al., 2014). We found that ergosterol peroxide was able to suppress TGF-β1-induced renal fibroblast proliferation and ECM production. Furthermore, the TGF-β1-stimulated phosphorylation of ERK1/2, p38, and the JNK pathway was inhibited by ergosterol peroxide.

Here, we further investigate the physiologically active compounds of *C. cicadae* by assessing the effects of HEA in the unilateral ureteric obstructive model, a model was widely used to study the molecular and cellular factors involved in renal fibrosis. Which is species- and strain independent and demonstrates changes that mimic the pathology of human progressive renal disease, and recapitulates the fundamental pathogenetic mechanisms that typify all forms of CKD in a relatively short time span (Eddy et al., 2012). We specifically examined the accumulation of fibrosis-related proteins and inflammatory cytokines. The impact of HEA was also determined on lipopolysaccharide (LPS)-induced inflammatory cytokine levels in a mouse macrophage cell line (RAW 264.7) and TGF-β1-induced fibroblast activation in a rat kidney fibroblast cell line (NRK-49F).

## Materials and Methods

### HEA Preparation

N6-(2-Hydroxyethyl) adenosine was purified from *C. cicadae* as described previously with some modifications (Lu et al., 2015). Briefly, extract of *C. cicadae* was produced by first adding powdered fungus to ethanol. After soaking overnight, the suspension was centrifuged, filtered, and concentrated under reduced pressure. An equal volume of hexane and ethyl acetate was added to a solution of the extract. After removing protein and polysaccharides from the aqueous layer with alcohol, HEA was eluted using an RP-C18 column (LiChroprep, Merck, United States) and purified by preparative thin-layer chromatography (Sephadex LH-20, GE Healthcare Bio-Sciences AB Sweden). The presence of HEA was confirmed by HPLC with an HEA standard.

### Animal Study

Male C57BL6/J mice, weighing 20–25 g were obtained from the SLAC Experimental Animal Center (Shanghai, China) and were housed at 22 ± 1°C, adherent to a 12-h light–dark cycle. After 1 week of adaptive feeding, mice were randomly divided into six groups (*n* = 6 each group): sham group (sham with vehicle treatment), sham+HEA (7.5 mg/kg) group (sham mice with 7.5 mg/kg HEA treatment), UUO group ( UUO with vehicle treatment ), and UUO+HEA (2.5 mg/kg), UUO+HEA (5 mg/kg), UUO+HEA (7.5 mg/kg) groups (UUO mice with 2.5, 5, or 7.5 mg/kg HEA treatment, respectively). And different doses of HEA (2.5, 5, and 7.5 mg/kg) were given by intraperitoneal injection 24 h before sham or UUO, and the treatment was continued for 14 days post-operatively. Renal fibrosis was induced by UUO as described previously (Zhao et al., 2016). Briefly, mice were anesthetized with isoflurane inhalation, and then UUO was achieved by double ligation of the left ureter with 3-0 silk through a left lateral incision. The same method was performed on sham-operated animals without ligation of the ureter. Two weeks after UUO, the mice were anesthetized and the obstructed kidney was harvested, prepared for histologic examinations and stored at -80°C for western blot analysis and cytokine assays. All procedures were performed according to a protocol approved by the Institutional Animal Care and Use Committee of Longhua Hospital, Shanghai University of Traditional Chinese Medicine, China.

### Histopathological Analyses

One half of the obstructed kidney tissue was fixed in buffered 4% paraformaldehyde for 24 h and then embedded in paraffin wax. To assess tubulointerstitial injury and fibrosis, 5-μm sections were stained separately with hematoxylin and eosin (H&E), Masson’s trichrome and picrosirius red. Tubular injury, characterized by tubular dilation and epithelial desquamation with interstitial expansions, was graded according to the extent of cortical involvement on a scale from 0 to 4 and assessed using a semi-quantitative scale as described in Kim et al. (2016). The semi-quantitative percentage of damaged area was scored as follows: 0, none; 0.5, <10%; 1, 10–25%; 2, 25–50%; 3, 50–70%; and 4, >75%. Image analysis for the quantification of fibrotic areas and positive area for TGF-β1 and α-SMA was performed by Image J (Bethesda, MD, United States) in 10 randomly selected microscopic fields per specimen.

### Immunofluorescent Staining

The antigen was retrieved from sections of formalin-fixed, paraffin-embedded tissue after dewaxing and rehydrating. Primary antibodies for inducible nitric oxide synthase (iNOS) and F4/80 or IL-10 and F4/80 double immunostaining, rabbit iNOS (Abcam, Cambridge, United Kingdom) or IL-10 (Santa Cruz Biotechnology, Dallas, TX, United States) monoclonal antibody and rat F4/80 monoclonal antibody (Abcam), anti-TGF-β1 (Santa Cruz Biotechnology), and anti-α-SMA (Sigma-Aldrich, St. Louis, MO, United States) were all 1:100 dilution and applied at the same time. Secondary antibodies included both FITC-conjugated goat anti-rabbit antibody and TRITC-conjugated goat-anti-rat antibody all purchased from Life Tech (Carlsbad, CA, United States).

### Cell Culture and Treatment

Mouse macrophage (RAW 264.7 cells; ATCC, Manassas, VA, United States) and rat renal fibroblast (NRK-49F; ATCC) cell lines were grown as monolayer cultures in Dulbecco’s modified Eagle medium (DMEM-F12, Sigma-Aldrich) supplemented with 10% heat-inactivated fetal bovine serum, penicillin (100 U/ml), and streptomycin (100 mg/ml) in an atmosphere of 5% CO_2_ and 95% air at 37°C. The medium was changed every 48 h. RAW 264.7 and NRK-49F cells were added to 24-well plates at a density of 2 × 10^5^ and 4 × 10^4^ cells/ml, respectively.

To investigate the effect of HEA on inflammatory and fibroblast activation, RAW 264.7 and NRK-49F cells were pretreated with HEA (5, 10, and 20 μg/ml) for 1 h, and then stimulated with LPS (1 μg/ml; Sigma-Aldrich) and TGF-β1 (2.5 ng/ml; Sigma-Aldrich) for 24 h.

### Cell Viability Assay

Effect of HEA on cell viability of NRK-49F and RAW 264.7 was determined by using the Cell Proliferation Reagent Kit I (MTT) (Roche, Basel, Switzerland) according to the manufacturer’s instructions. Cells were treated with serial concentrations of HEA (0, 5, 10, and 20 μg/ml) for 24 h and then viability was assessed.

### Quantitative Real-Time PCR Analysis

Total RNA was extracted from kidneys using Trizol reagent (Thermo Fisher Scientific, Waltham, MA, United States) and cDNA was synthesized with a SuperScript cDNA Synthesis Kit (Thermo Fisher Scientific). Quantitative real-time PCR was performed using a Fast SYBR Green mix kit (Qiagen, Valencia, CA, United States) on the ABI-StepOnePlus Sequence Detection System (Applied Biosystems, Foster City, CA, United States). The primer sequences used in this study are listed in **Table 1**. Cycle threshold (CT) values of each mRNA were normalized to those of GAPDH. Fold differences were determined using the 2^-ΔΔCT^ method and analyzed relative to those of WT mice in the sham group.

**Table 1:** Sequences of qRT-PCR primers used in this study.

Gene	Primer sequence (5′ > 3′)
TGF-β1	Forward: CGTCAGACATTCGGGAAGCA
	Reverse: TGCCGTACAACTCCAGTGAC
Collagen I	Forward: TGCCGTGACCTCAAGATGTG
	Reverse: CACAAGCGTGCTGTAGGTGA
α-SMA	Forward: GTTTCTCGCACGTCTCCTCT
	Reverse: CAGGCAGTTCGTAGCTCTTC
Fibronectin	Forward: ACTCCTTGCTGGTGTCATGG
	Reverse: GGAAGGGTAACCAGTTGGGG
TNF-α	Forward:AGGCACTCCCCCAAAAGATG
	Reverse: CCACTTGGTGGTTTGTGAGTG
IL-1β	Forward: ATGCCACCTTTTGACAGTGATG
	Reverse: GAAGGTCCACGGGAAAGACA
IL-6	Forward: GCCTTCTTGGGACTGATGCT
	Reverse: CTGCAAGTGCATCATCGTTGT
IL-10	Forward: GCCGGGAAGACAATAACTGC
	Reverse: AAGGCTTGGCAACCCAAGTA
GAPDH	Forward: CATCTTCCAGGAGCGAGACC
	Reverse: CTCGTGGTTCACACCCATCA


### Western Blot Analysis

Cells were lysed in buffer (20 mM Tris-HCl pH 8, 150 mM NaCl) containing a protease inhibitor. Protein concentrations were determined using a BCA Protein Assay Kit. A total of 20–30 μg of proteins were separated using 12% sodium dodecyl sulfate-polyacrylamide gel electrophoresis and then transferred to a nitrocellulose membrane. The membrane was blocked with 5% (w/v) non-fat milk in Tris-buffered saline with 20% TWEEN-20 (TBS-T). Membranes were then incubated in primary antibodies overnight: anti-TGF-β1 (Santa Cruz Biotechnology), Anti-α-SMA (Sigma-Aldrich), Anti-collagen I (Santa Cruz Biotechnology), Anti-fibronectin (Abcam), Anti-iNOS (Abcam), Anti-IL-10 (Santa Cruz Biotechnology), Anti-p-Smad2 (Cell Signaling Technology), Anti-Smad2 (Santa Cruz Biotechnology), Anti-p-Smad3 (Cell Signaling Technology), Anti-Smad3 (Santa Cruz Biotechnology), Anti-Smad7 (Santa Cruz Biotechnology), and Anti-p-NF-κB, Anti-NF-κB, Anti-p-IκBα, Anti-IκBα (Cell Signaling Technology). After incubation, anti-rabbit IgG and anti-goat IgG (Santa Cruz Biotechnology) were used to detect proteins. The membranes were visualized using an enhanced chemiluminescence detection (ECL) kit (Amersham Pharmacia Biotech, Piscataway, NJ, United States). Densitometric analysis was performed using ImageJ software^1^.

### Enzyme-Linked Immunosorbent Assay (ELISA)

Enzyme-linked immunosorbent assay (ELISA) kits were used to detect mouse TNF-α, IL-6, IL-1β and IL-10 (R&D Systems, Minneapolis, MN, United States) in kidney tissue homogenates according to manufacturer’s instructions. Proteins were detected in samples using horseradish peroxidase (HRP)-conjugated reagent and incubated for 1 h at 37°C. After washing, tetramethylbenzidine (TMB) was added and the chromogenic reaction was terminated with Stop Solution. Absorbance was measured at 450 nm with an iMark^TM^ Microplate Reader (Bio-Rad, Tokyo, Japan). Protein concentrations were determined in the kidney tissue homogenates using a BCA Protein Assay Kit.

### Statistical Analysis

All data are presented as the mean ± SEM. All experiments were replicated three times. Statistical analyses were conducted using one-way ANOVA with the Hochberg test and two-sample *t*-tests. All tests were conducted using SPSS 17.0 software (SPSS, Inc., Chicago, IL, United States) and A *P*-value < 0.05 was considered statistically significant.

## Results

### Injury of Kidney Tissue in UUO Mice Ameliorated by Intraperitoneal Administration of HEA

**FIGURE 1:**
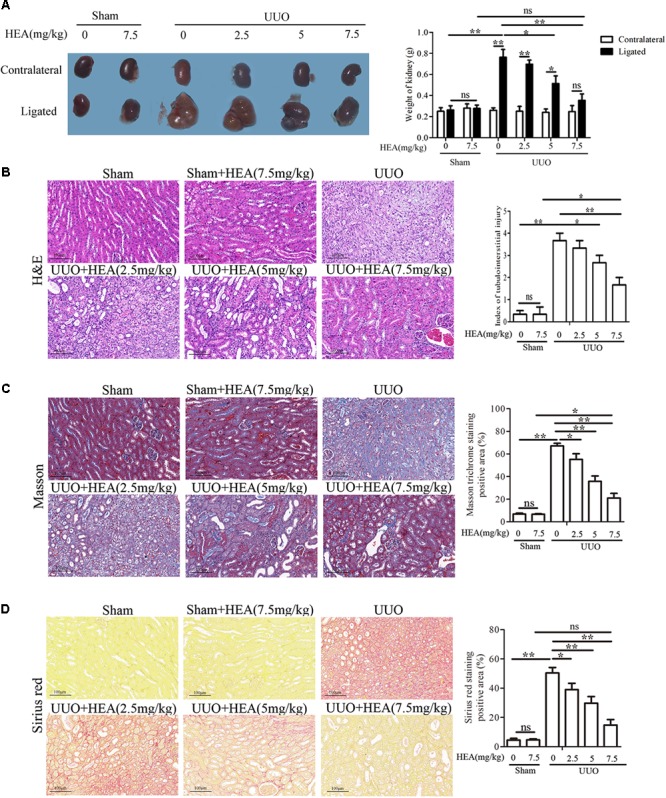
Effect of intraperitoneal administration of HEA on kidney tissue in UUO mice. **(A)** Two weeks after the operation, the kidneys were weighed; the enlargement of kidneys was improved after HEA treatment. **(B)** H&E staining revealed improved integrity of the renal parenchymal cells in the ligated kidneys from UUO mice after different doses of HEA treatment, the stained slides were scored by the semi-quantitative percentage of damaged area as follows: 0, none; 0.5, <10%; 1, 10–25%; 2, 25–50%; 3, 50–70%; and 4, >75%. **(C)** Masson staining showed massive collagen deposition (blue) in the ligated kidneys from UUO mice, and less collagen was observed after HEA treatment. **(D)** Sirius red-stained kidney sections also indicated that HEA treatment could strikingly decrease collagen deposition (red) in UUO (Original magnification ×200). The area of connective tissue was assessed and quantified by Image J. HEA (2.5 mg/kg), HEA (5 mg/kg), and HEA (7.5 mg/kg): different dose of HEA treatment (2.5, 5, and 7.5 mg/kg, respectively). The data are presented as the means ± SEM. ^∗^*P* < 0.05 and ^∗∗^*P* < 0.01. Scale bar = 100 μM.

Severe hydronephrosis with marked loss of renal parenchyma usually occurs in kidneys within 14 days of performing UUO (Pang et al., 2010). Therefore, we evaluated the effects of HEA on ligated and contralateral kidneys in UUO and sham-operated mice 2 weeks post-operatively. Sham-operated mice were used inthe comparison to account for any compensatory changes in the contralateral kidney. We found that the enlargement in hydronephrotic kidneys induced by UUO (263.33 ± 65.71 in sham vs. 763.33 ± 125.32 in UUO, mg, *P* < 0.01) was significantly reduced after HEA treatment [763.33 ± 125.32 in UUO vs. 351.66 ± 92.43 in UUO+HEA (7.5 mg/kg), mg, *P* < 0.01] and this improvement was dose-dependent (2.5–7.5 mg/kg) (**Figure 1A**). In addition, H&E staining combined with semi-quantitative scoring of kidney tissue revealed that HEA improved the integrity of the renal parenchymal cells in the ligated kidneys from UUO mice [0.42 ± 0.34 in sham vs. 3.67 ± 0.47 in UUO vs. 1.75 ± 0.80 in UUO+HEA (7.5 mg/kg), *P* < 0.01] (**Figure 1B**). Moreover, Masson and Sirius red staining confirmed that HEA treatment decreased the deposition of collagen in the ligated kidneys of UUO mice (**Figures 1C,D**). Significantly less staining was observed in HEA-treated mice kidneys following UUO (*P* < 0.01). These results indicate that HEA can prevent the hydronephrosis and ameliorate injury of kidney tissue in a renal interstitial fibrosis model induced by UUO in mice.

### HEA Reduces the Accumulation of Fibrosis-Related Proteins and Inflammatory Cytokine in the Ligated Kidneys of UUO Mice

**FIGURE 2:**
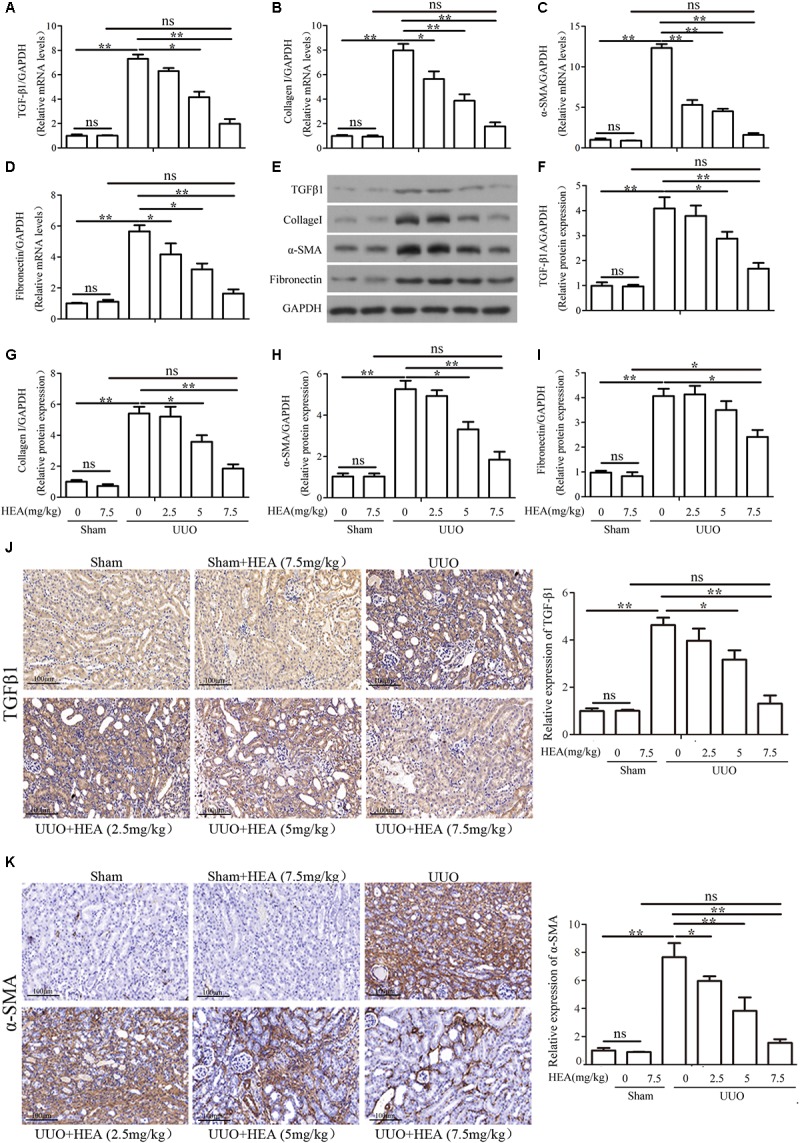
Effect of HEA treatment on fibrosis-related gene and protein expression in ligated kidneys. **(A–D)** Relative mRNA levels of TGF-β1, α-SMA, collagen I, and Fibronectin expression in kidneys detected by PCR. **(E)** Representative photographs showing protein expression of TGF-β1, α-SMA, collagen I and Fibronectin in the ligated kidney by western blot analysis. **(F–I)** Semi-quantitative analyses versus **(E)**. **(J,K)** The expression of TGF-β1 and α-SMA in ligated kidneys detected by immunohistochemistry. The relative results showed that HEA treatment reduced TGF-β1, α-SMA, collagen I and Fibronectin levels in the left ligated kidney. The data are presented as the means ± SEM. ^∗^*P* < 0.05 and ^∗∗^*P* < 0.01. Scale bar = 100 μM.

We next assessed the impact of HEA on the level of fibrosis induced by UUO in the kidneys of the mice. Relative mRNA levels of the fibrosis-related genes TGF-β1, α-SMA, collagen I, and Fibronectin in ligated kidneys were measured by PCR (**Figures 2A–D**) and corresponding protein levels were analyzed with western blotting (**Figures 2E–I**). The mRNA expression and protein levels of TGF-β1, α-SMA, collagen I, and Fibronectin in ligated kidneys were all significantly increased (*P* < 0.01) in the ligated kidney but levels decreased dose-dependently in response to HEA administration (*P* < 0.05). Very little change was observed in the mRNA expression or levels of these proteins in the kidneys of sham-operated mice (*P* > 0.05). We also detected the presence of TGF-β1 and α-SMA in ligated kidney sections by immunohistochemistry (**Figures 2J,K**). The intensity of TGF-β1 and α-SMA staining reflected the dose of HEA. With the most intense staining found in untreated ligated kidneys and the least in the sham-operated kidney treated with the highest dose of HEA.

**FIGURE 3:**
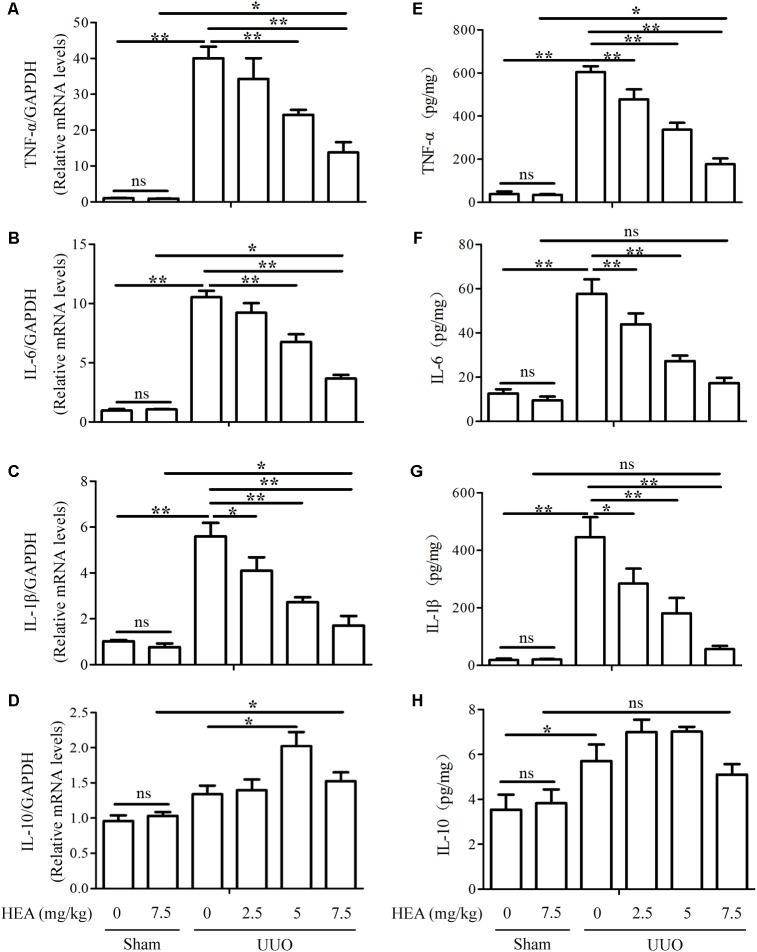
Effect of HEA on inflammatory cytokines in ligated kidneys. **(A–D)** HEA treatment significantly reduced the mRNA levels of the inflammatory cytokines TNF-α, IL-6, and IL-1β, while increasing IL-10. **(E–H)** Representative ELISA for inflammatory markers (TNF-α, IL-6, IL-1β, and IL-10) in ligated kidney tissue. The data are presented as the means ± SEM. ^∗^*P* < 0.05 and ^∗∗^*P* < 0.01.

We also assessed the levels of the inflammatory cytokines TNF-α, IL-6, IL-1β, and IL-10 in ligated kidneys with and without HEA treatment. The levels of TNF-α, IL-6, and IL-1β mRNA expression were significantly elevated in ligated kidneys compared with sham-operated controls (*P* < 0.01) but these elevated levels were significantly reduced by HEA in a dose-dependent manner (*P* < 0.01) (**Figures 3A–C**). Interestingly, the UUO had no significant effect on the mRNA expression of IL-10 (*P* > 0.05), whereas HEA increased the level of expression (**Figure 3D**). However, HEA treatment increased the expression of IL-10 in ligated kidneys, with the highest expression observed at a dose of 5 mg/kg (*P* < 0.05). This effect was not dose-dependent as HEA at 7.5 mg/kg produced a lower expression of IL-10 mRNA than 5 mg/kg. In contrast, HEA at 7.5 mg/kg did not increase expression of IL-10 mRNA in sham-operated kidneys. Therefore, the increased expression of IL-10 mRNA was probably in response to the UUO rather than the HEA treatment. A similar pattern was detected in kidney homogenates by ELISA assays. The significantly elevated levels of TNF-α [39.28 ± 15.23 in sham vs. 604.4 ± 38.34 in UUO vs. 176.81 ± 38.04 in UUO+HEA (7.5 mg/kg), pg/mg, *P* < 0.01], IL-6 [12.54 ± 2.81 in sham vs. 57.61 ± 9.36 in UUO vs. 17.22 ± 3.51 in UUO+HEA (7.5 mg/kg), pg/mg, *P* < 0.01], and IL-1β [18.24 ± 7.99 in sham vs. 445.66 ± 98.70 in UUO vs. 56.33 ± 16.18 in UUO+HEA (7.5 mg/kg), pg/mg, *P* < 0.01] found in ligated kidneys decreased with increasing dose of HEA (**Figures 3E–G**) whereas levels of IL-10 increased and were at the highest levels at 2.5–5 mg/kg HEA (**Figure 3H**). The results indicate that HEA treatment prevents the accumulation of fibrosis-related proteins and inflammatory cytokines associated with renal interstitial fibrosis.

### HEA Effects on Macrophage Phenotype in a Renal Interstitial Fibrosis Mouse Model

**FIGURE 4:**
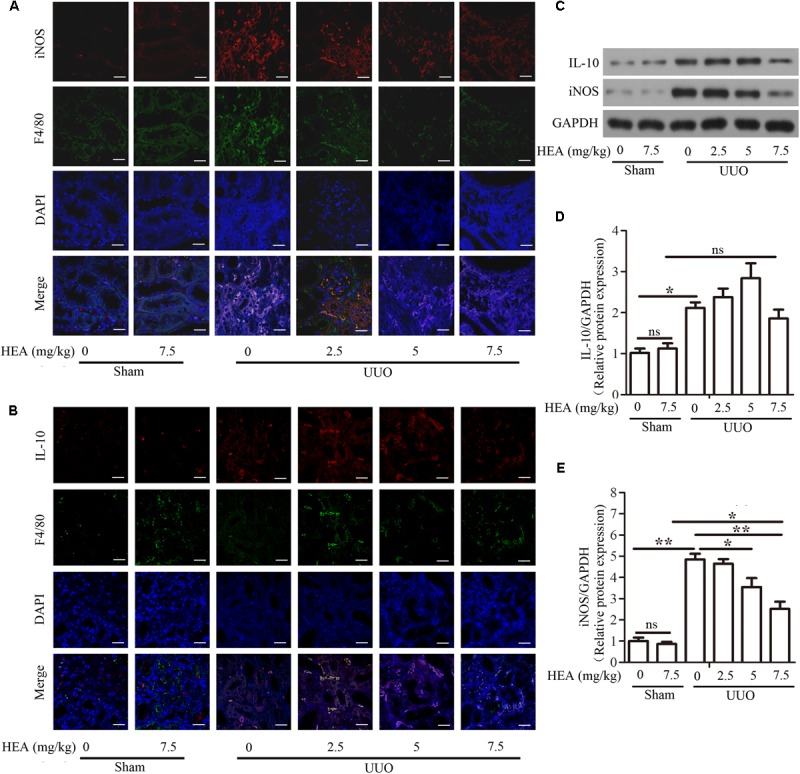
Effect of HEA on macrophage phenotype in UUO mice. Representative immunofluorescence images of F4/80-labeled macrophages (green) from the sham and UUO groups. M1 macrophages were immunostained with inducible nitric oxide synthase (iNOS) and M2 macrophages were immunostained with IL-10. DAPI labels in the nucleus (blue) (Magnification ×600). **(A)** HEA blocked the accumulation of M1 macrophages and induced the accumulation of M2 macrophages in UUO kidneys **(B)** iNOS and IL-10 expression were examined by western blot **(C)** and semi-quantified by normalization to GAPDH **(D,E)**. The data are presented as the means ± SEM. ^∗^*P* < 0.05 and ^∗∗^*P* < 0.01. Scale bar = 20 μM.

As the M1/M2 macrophage ratio is an important stage in the development of renal interstitial fibrosis, we assessed the effects of HEA on the production of M1 macrophages by immunostaining sections with iNOS and M2 macrophages by immunostaining sections with IL-10. Simultaneously, we used F4/80 antigen to detect interstitial resident macrophages, which are not found in normal mesangium. We found that HEA could block the accumulation of M1 macrophages, and induced the accumulation of M2 macrophages in ligated kidneys (**Figures 4A,B**). Untreated ligated sections clearly show positive detection of F4/80 antigen, whereas F4/80 antigen is significantly reduced in sections treated with the highest dose of HEA. Similarly, levels of iNOS are highest in untreated sections whereas levels of IL-10 are complex with increased dose of HEA, similar to the above. And the dose-independent levels of IL-10 in response to HEA treatment indicate that the anti-inflammatory effect of high-dose HEA may be to regulate IL-10 levels eventually becomes normal. Western blotting further confirmed the levels of iNOS and IL-10 corresponding to the dose of HEA (**Figures 4C–E**).

### HEA Inhibits the TGF-β1/Smad and NF-κB Signaling Pathway

**FIGURE 5:**
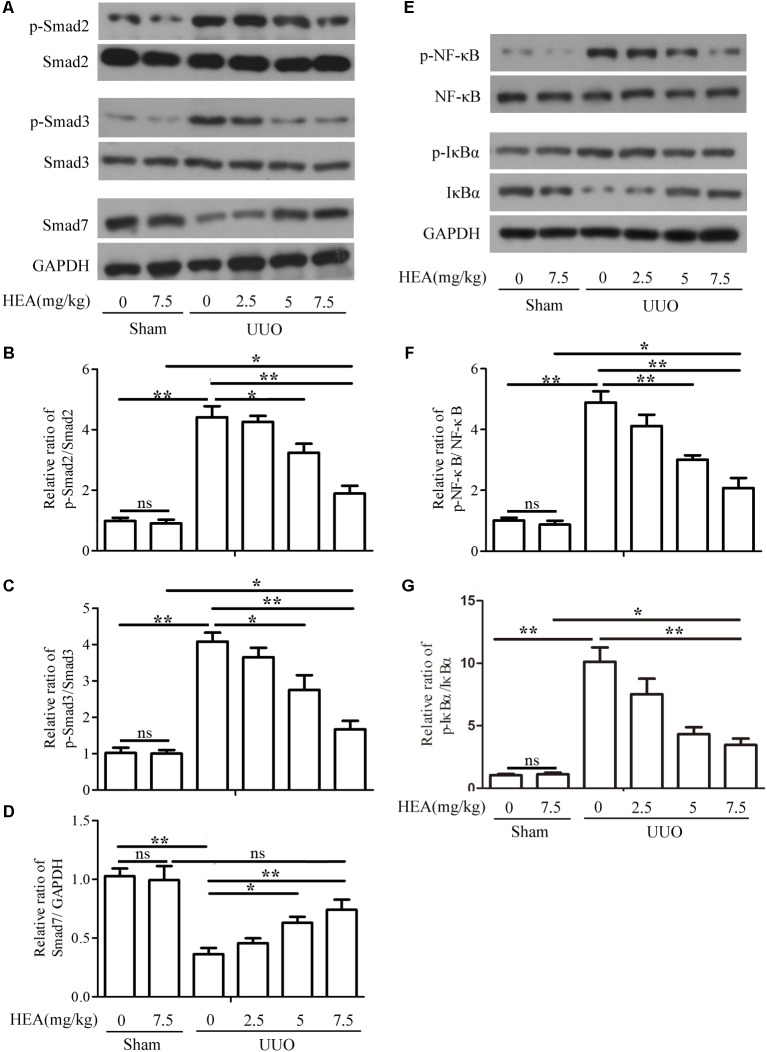
Effect of HEA on UUO-induced TGF-β1/Smad and NF-κB signaling pathways. **(A)** Phospho-Smad2/Smad2, phospho-Smad3/Smad3, and Smad7 expression in kidney tissue from sham- and UUO-operated mice treated with vehicle (Veh) or HEA were evaluated by western blotting. **(B–D)** Data from densitometric analysis of phospho-Smad2, phospho-Smad3, and Smad7 are presented as the relative ratio of each protein to Smad2, Smad3, and GAPDH. The relative ratio measured in the kidneys from sham-operated mice treated with Veh is arbitrarily presented as 1. **(E)** Representative western blot for p-NF-κB, NF-κB, p-IκBα, IκBα, and GAPDH. **(F,G)** The ratio of p-NF-κB normalized to NF-κB protein levels, and the ratio of p-IκBα normalized with IκBα. The data are presented as the means ± SEM. ^∗^*P* < 0.05 and ^∗∗^*P* < 0.01.

To evaluate the impact of HEA on the TGF-β1/Smad and NF-κB signaling pathway, we analyzed components of both pathways in sham- and UUO-operated mice treated with vehicle or HEA. Phospho-Smad2/Smad2, phospho-Smad3/Smad3, and Smad7 expression were evaluated by western blotting (**Figure 5A**) and presented graphically using densitometric analysis (**Figures 5B–D**). The activity of Smad2 and Smad3 is significantly increased by UUO (*P* < 0.01) whereas Smad7 expression is significantly reduced (*P* < 0.01). The activity of Smad2 and Smad3 is reduced by HEA dose-dependently following UUO (*P* < 0.05). In contrast, Smad7 expression significantly increases in response to HEA treatment (*P* < 0.05). Representative western blots for p-NF-κB, NF-κB, p-IκBα, IκBα, and GAPDH are shown in **Figure 5E**. The ratio of p-NF-κB normalized to NF-κB protein levels, and the ratio of p-IκBα, IκBα normalized to GAPDH protein levels are shown in **Figures 5F,G**. The activity of NF-κB and IκBα is significantly increased in ligated kidneys (*P* < 0.01) but reduced dose-dependently in response to HEA treatment (*P* < 0.01). Taken together, these results indicate that the TGF-β1/Smad and NF-κB signaling pathways are activated in response to renal injury induced by UUO, whereas, HEA treatment suppresses the activation of both pathways.

### TGF-β1 or LPS-Induced TGF-β1/Smad and NF-κB Pathways Are Modulated by HEA *in vitro*

**FIGURE 6:**
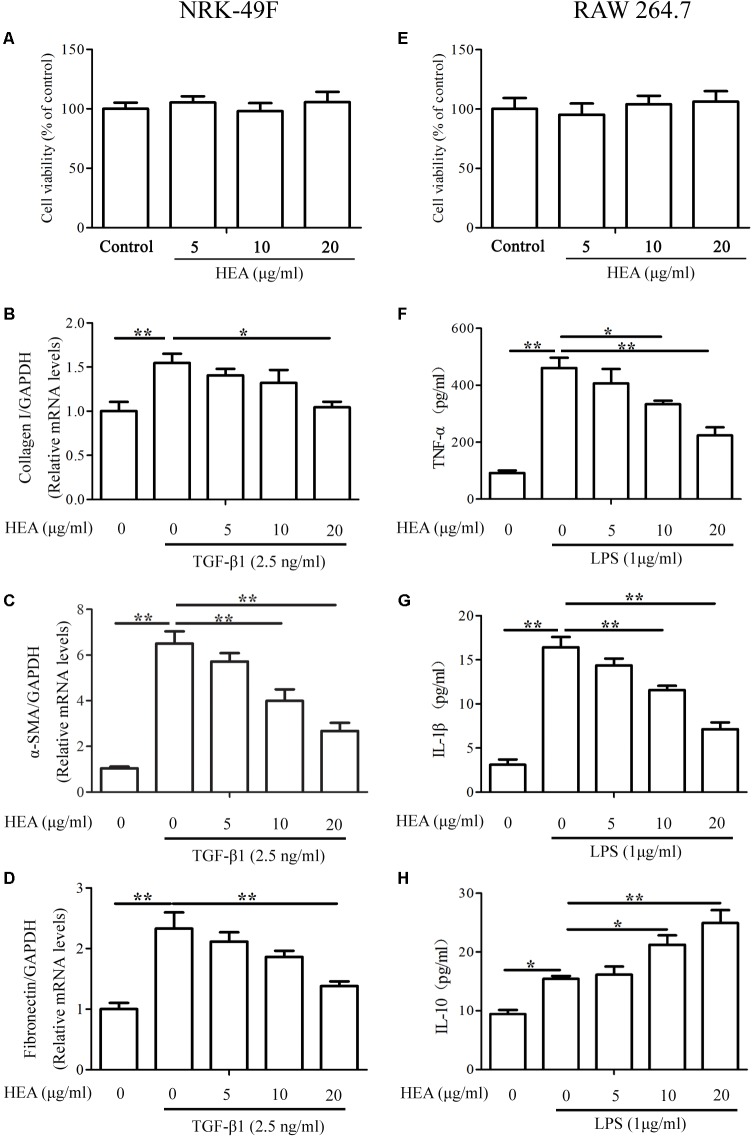
Effect of HEA on cell viability, fibrosis, and inflammation in the *in vitro* assay. **(A,E)** Cell viability was assessed with a MTT assay after NRK-49F and RAW 264.7 cells were incubated with 5, 10, and 20 μg/ml HEA for 24 h; 0 μg/ml HEA was used as the control group. NRK-49F and RAW 264.7 cells were pretreated with HEA for 1 h and then stimulated by transforming growth factor-β (TGF-β; 2.5 ng/ml) and lipopolysaccharide (LPS; 1 μg/ml) 24 h. After a 24 h incubation, the NRK-49F cells were analyzed by PCR for collagen I **(B)**, α-SMA **(C)**, and Fibronectin **(D)**. RAW 264.7 cells were analyzed by ELISA for TNF-α **(F)**, IL-1β **(G)**, and IL-10 **(H)**, respectively. The data are presented as the means ± SEM. ^∗^*P* < 0.05 and ^∗∗^*P* < 0.01.

The abilities of HEA to ameliorate the production of ECM and inflammatory cytokines were also assessed by *in vitro* experiments using the cell lines NRK-49F and RAW 264.7. Serial concentrations of HEA used in this study did not induce cytotoxicity in either NRK-49F or RAW 264.7 cells (**Figures 6A,E**). The expression of the ECM-related proteins collagen I, α-SMA and Fibronectin were assessed in NRK-49F cells by PCR (**Figures 6B–D**) and levels of TNF-α, IL-1β, and IL-10 were assessed in RAW 264.7 cells by ELISA (**Figures 6F–H**). Results indicate that TGF-β and LPS initiate the production of ECM in NRK-49F cells because levels of collagen I, α-SMA and Fibronectin are all increased after stimulation. However, the levels of ECM production, indicated by expression of collagen I (*P* < 0.05), α-SMA (*P* < 0.01) and Fibronectin (*P* < 0.01), are significantly reduced by treatment with HEA dose-dependently. Similarly, TGF-β and LPS stimulate increased levels of TNF-α, IL-1β and IL-10. HEA treatment reduces the elevated levels of TNF-α and IL-1β and increases IL-10 secretion dose-dependently.

**FIGURE 7:**
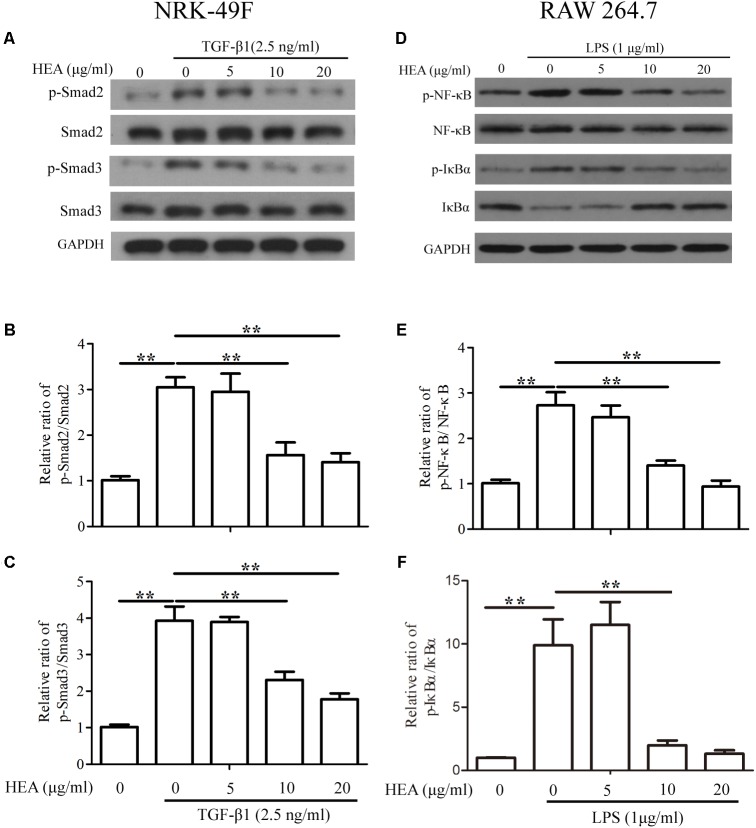
Effect of HEA on transforming growth factor (TGF)-β1 or lipopolysaccharide (LPS)-induced TGF-β1/Smad and NF-κB signaling pathway *in vitro*. NRK-49F and RAW 264.7 cells were pretreated with HEA for 1hn and then stimulated by TGF-β1 (2.5 ng/ml) and LPS (1 μg/ml). After 24 h incubation, both cell lines were subjected to western blot analysis for P-Smad2/Smad2, P-Smad3/Smad3 **(A)** and p-NF-κB/NF-κB, p-IκBα/IκBα **(D)**. **(B,C)** Data from densitometric analysis of P-Smad2 and P-Smad3 are presented as the relative ratio of each protein to Smad2 and Smad3. **(E–F)** The ratio of p-NF-κB normalized to NF-κB protein levels, and the ratio of p-IκBα normalized to IκBα. The data are presented as the means ± SEM. ^∗^*P* < 0.05 and ^∗∗^*P* < 0.01.

Under the same experimental conditions (pretreated with HEA and then stimulated by TGF-β1 or LPS), we assessed the levels of P-Smad2/Smad2, P-Smad3/Smad3 in NRK-49F cells and p-NF-κB/NF-κB, p-IκBα/IκBα in RAW 264.7 cells by western blotting and densitometric analysis. The activity of Smad2 and Smad3 was increased in NRK-49F cells in response to stimulation by TGF-β1 and LPS but was significantly decreased by pretreatment with HEA dose-dependently (*P* < 0.01) (**Figures 7A–C**). Similar results were observed with NF-κB but the activity of IκBα was reduced upon stimulation by TGF-β1 and LPS and increased on treatment with HEA (*P* < 0.01) (**Figures 7D–F**). These combined results indicate that HEA can suppress TGF-β1 or LPS-induced TGF-β1/Smad and NF-κB signaling pathways *in vitro*.

## Discussion

Chronic kidney disease, characterized by the accumulation of ECM proteins in the glomerulus and interstitium, gives rise to renal interstitial fibrosis through the promotion of an aberrant wound-healing response involving tubular epithelial cells, myofibroblasts, fibrocytes, and immune cells (Guiteras et al., 2016). In the present study, we have investigated whether the medicinal properties of HEA, an active constituent found in the entomopathogenic fungus *C. cicadae*, could alleviate the progress of renal interstitial fibrosis in a UUO mouse model. We found that intraperitoneal administration of HEA ameliorated inflammation, reduced the accumulation of fibrosis-related proteins and inflammatory cytokines, rebalanced the M1/M2 macrophage ratio, and suppressed the TGF-β1/Smad and NF-κB signaling pathway in ligated kidneys.

In UUO-operated mice, we observed an increase of IL-10 expression in response to kidney ligation. The major source of IL-10 in a normal adult kidney is mesangial cells, which contribute to the secretion and maintenance of the ECM (Sinuani et al., 2006). A correlation exists between the degree of macrophage infiltration and the severity of chronic kidney disease in humans suggesting that macrophages may have an effector function in the progression of the disease (Cao et al., 2018). The M1 subpopulation of macrophages is known to have a pathogenic function in renal inflammation whereas the M2 macrophage subpopulation is involved in the resolution of inflammation and injury repair. In this study, we detected levels of M1 macrophages using the markers F4/80 antigen and iNOS. M2 macrophages are known to secrete anti-inflammatory cytokines such as IL-10 and TGF-β, therefore, we assessed increased M2 activity by detecting IL-10. IL-10 and TGF-β are thought to coregulate the production of inflammatory cytokines, chemokines, and nitric oxide (Sinuani et al., 2013). The increase in the level of IL-10 after HEA treatment in this study suggests an increase in M2 activity. However, the alleviation of fibrotic characteristics was not only due to the stimulation of M2 proliferation directly by HEA because the kidney tissues of sham-operated mice administered with the highest dose of HEA do not exhibit an increase in IL-10 levels. Moreover, the highest concentration of HEA did not correspond to the highest level of IL-10 expression in some of our experiments. Therefore, the increase in levels of IL-10 was probably due to other factors associated with the renal injury and relatively uninfluenced by the intraperitoneal administration of HEA. However, the renal injury alone did not lead to increased levels of IL-10, therefore, the increased levels of IL-10 must be associated, in some way, with the repair process induced by HEA administration. Activated M2 macrophages can be further categorized into three subgroups: M2a, induced by IL-4 and/or IL-13, are involved in wound-healing; M2b are induced by immune complexes; and M2c, induced by IL-10, TGF-β or glucocorticoid, have anti-inflammatory effects (Anders and Ryu, 2011). This indicates that HEA may induce an anti-inflammatory effect and explain why the levels of IL-10 are reduced at higher doses of HEA when kidney inflammation subsides. Certainly, further investigations are warranted to deduce the specific role of IL-10 in this process.

The TGF-β1/Smad and NF-κB signaling pathways are known to be major contributors to renal fibrosis (Lan, 2011). Our studies specifically examined whether HEA could influence the NF-κB and TGF-β1/Smad signaling pathway. Levels of Smad7 were reduced in ligated kidneys compared to the sham-operated control but increased dose-dependently in response to HEA treatment. Whereas, phosphorylation of Smad2 and Smad3 were increased in ligated kidneys but the activity of Smad2 and Smad3 were reduced in HEA-treated mice. The degradation and ubiquitination of Smad7 appear to be a characteristic of UUO kidneys and is thought to play a pathogenic role in the progression of tubulointerstitial fibrosis (Fukasawa et al., 2004). Levels of the Smad ubiquitination regulatory factors are elevated in UUO kidneys and interact with Smad7, therefore, preventing Smad7 from suppressing TGF-β signaling. Smad7 is thought to inhibit TGF-β signaling by associating with activated TβRI and preventing the activation of Smads (Fukasawa et al., 2004). Smad7 can also influence NF-κB activation. Inhibition of NF-κB activation by Smad7-gene therapy was found to reduce renal injury in murine models (Ng et al., 2005). Here, increased NF-κB and TGF-β1/Smad signaling activation following UUO was inhibited by HEA treatment. Thereby, preventing the upregulation of inflammatory factors and ECM-related proteins associated with renal interstitial fibrosis.

### Study Limitations

Our current study has some limitations that deserve to be mentioned. First, lack of the preventative nature of the study design used. Another issue is no comparison between HEA and other components of *C. cicadae* or currently-used medications for CKD/kidney fibrosis.

In addition, adenosine, as an important intermediate in the biosynthetic pathway for generating adenosine triphosphate (ATP), can be released from cells to act physiologically as extracellular messengers or pathologically as danger signals. The extracellular adenosine acts as a stress hormone, stimulate membrane receptors in the P2 and P1 family to modulate numerous physiological processes including chronic kidney disease (Menzies et al., 2017). Our study, HEA, as a derivative of adenosine, had been found to exert its pharmacological activity via acting on adenosine receptor (Fang et al., 2016). And kidney tissue injury causes a localized increase in ATP concentration, and sustained activation of purinergic signaling can lead to renal glomerular, tubular, and vascular cell damage (Goncalves et al., 2006; Komada et al., 2014). And tissue fibroblasts express multiple purinergic receptors and respond to extracellular nucleotides by activating key pathways for the production of ECM (Solini et al., 2005; Chen et al., 2012; Lu and Insel, 2013). Our results also confirm that the increase of ECM-related proteins in response to TGF-β and LPS stimulation are reduced with HEA treatment and can prevent the production of ECM-related proteins and the accumulation of cytokines that give rise to fibrosis. Therefore, whether there is mutual regulation between purinergic signaling mechanism and HEA or other active components of *C. cicadae* is worthy of further study.

## Conclusion

This study, HEA has a beneficial effect on UUO-induced tubulointerstitial fibrosis by suppression of inflammatory and renal fibroblast activation via modulation of the NF-κB and TGF-β1/Smad signaling pathway. It provides further evidence that the medicinal properties of *C. cicadae*, which include the active components HEA and ergosterol peroxide, could be useful in the treatment of renal interstitial fibrosis.

## Author Contributions

RZhe and RZhu performed the experiments and drafted the article. XuL, XiL, LS, and YC helped to perform the research, contributed new reagents, analytic tools, and analyzed the data. YZ and YD designed the research, analyzed the data, edited and revised the manuscript, and revised and approved the final version of the manuscript.

## Conflict of Interest Statement

The authors declare that the research was conducted in the absence of any commercial or financial relationships that could be construed as a potential conflict of interest. The reviewer JY and handling Editor declared their shared affiliation at the time of the review.

## Footnotes

1 http://rsbweb.nih.gov/ij
